# Four Promoters of IRF5 Respond Distinctly to Stimuli and are Affected by Autoimmune-Risk Polymorphisms

**DOI:** 10.3389/fimmu.2013.00360

**Published:** 2013-11-07

**Authors:** Daniel N. Clark, R. Daniel Read, Vera Mayhew, Stephen C. Petersen, Lissenya B. Argueta, Lance A. Stutz, Rodney E. Till, Sean M. Bergsten, Brandon S. Robinson, Douglas G. Baumann, J. Casey Heap, Brian D. Poole

**Affiliations:** ^1^Department of Microbiology and Molecular Biology, Brigham Young University, Provo, UT, USA

**Keywords:** IRF5, alternative promoters, autoimmune disease risk, interferon, systemic lupus erythematosus

## Abstract

**Introduction:** Autoimmune diseases such as systemic lupus erythematosus, rheumatoid arthritis, and multiple sclerosis affect millions of people worldwide. Interferon regulatory factor 5 (IRF5) contains polymorphisms associated with these autoimmune diseases. Two of these functional polymorphisms are found upstream of the IRF5 gene. rs2004640, which is a single nucleotide polymorphism and the CGGGG insertion/deletion (indel) were studied. IRF5 uses four different promoters for its four first exons: 1A, 1B, 1C, and 1D. Each promoter was analyzed, including functional differences due to the autoimmune-risk polymorphisms.

**Results:** IRF5 promoters were analyzed using ChIP-Seq data (ENCODE database) and the FactorBook database to define transcription factor binding sites. To verify promoter activity, the promoters were cloned into luciferase plasmids. Each construct exhibited luciferase activity. Exons 1A and 1D contain putative PU.1 and NFkB binding sites. Imiquimod, a Toll-like receptor 7 (TLR7) ligand, was used to activate these transcription factors. IRF5 levels were doubled after imiquimod treatment (*p* < 0.001), with specific increases in the 1A promoter (2.2-fold, *p* = 0.03) and 1D promoter (2.8-fold, *p* = 0.03). A putative binding site for p53, which affects apoptosis, was found in the promoter for exon 1B. However, site-directed mutagenesis of the p53 site showed no effect in a reporter assay.

**Conclusion:** The IRF5 exon 1B promoter has been characterized, and the responses of each IRF5 promoter to TLR7 stimulation have been determined. Changes in promoter activity and gene expression are likely due to specific and distinct transcription factors that bind to each promoter. Since high expression of IRF5 contributes to the development of autoimmune disease, understanding the source of increased IRF5 levels is key to understanding autoimmune etiology.

## Introduction

Alternative splicing is a method of making different transcripts from one genomic region. One type of alternative splicing involves the use of multiple first exons. This is termed alternative promoter splicing, since each first exon must have its own promoter. Alternative promoter splicing occurs in around half of human genes (1).

The gene interferon regulatory factor 5 (IRF5) is a transcription factor which controls immune signaling, cytokine expression, the cell cycle, and apoptosis (2–5). It exhibits alternative promoter splicing and has four different first exons that are currently known. The start codon for IRF5 is in exon 2, therefore the use of different first exons does not directly alter the protein sequence. Instead the four alternative promoters are four pathways to make the same protein. The first exons are 1A, 1B, 1C, and 1D.

**Figure 1:**
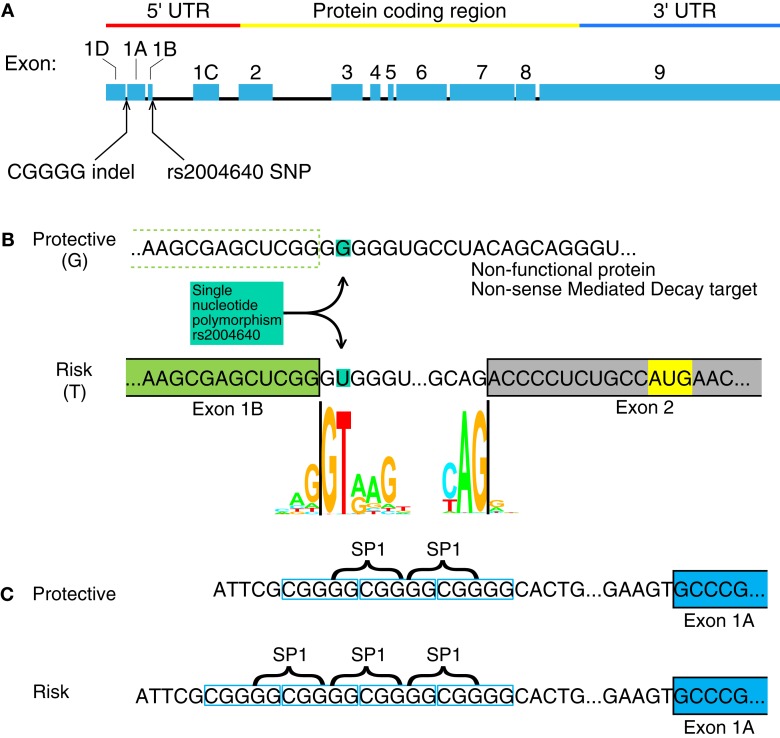
**Interferon regulatory factor 5 mRNA and the position of the rs2004640 SNP and CGGGG indel**. **(A)** rs2004640 is at the splice acceptor site for exon 1B, and the CGGGG indel is 64 bp upstream from the transcription start site for exon 1A. The genomic region of IRF5 is drawn to scale, but with introns reduced in size 10:1. The protein coding and untranslated regions are shown above. Only one first exon is used per RNA transcript; therefore each first exon corresponds to the untranslated region for that transcript. **(B)** The position of the rs2004640 SNP on pre-mRNA. Before splicing, the messenger RNA has either a U (encoded by the risk T allele) or G. The colored letters shown in the WebLogo (the nucleotide stacks of varying heights represent the consensus recognition sites for the spliceosome. The height of the stack represents how often those nucleotides are found at that position, and thus the high GT represents a strong preference for recognizing GT at the intron boundary. This matches in the risk T allele (GT at the intron boundary), but not the protective allele (GG at the intron boundary). A person homozygous for the protective allele cannot splice IRF5 mRNA that begins with exon 1B. Instead of a functional protein, the resultant mRNA would encode a non-functional protein and be targeted for non-sense mediated decay. Splice junction WebLogos are from Stephens and Schneider (7). (**C)** The CGGGG indel is an insertion/deletion of a CGGGG repeat upstream of exon 1A, and it is part of exon 1A’s promoter. When there are four copies, additional SP1 transcription factors (which bind to GGCGG) can bind to the promoter, altering transcription levels. UTR, untranslated region; SNP, single nucleotide polymorphism.

The IRF5 gene contains several GWAS-identified polymorphisms associated with autoimmune diseases. Among them, most do not have an assumed effect. Although IRF5 contains several polymorphisms associated with autoimmunity, only four have been identified as functional polymorphisms (6). Two of these are in the promoter or untranslated regions of IRF5 where the polymorphisms may have a direct effect on IRF5 expression: a single nucleotide polymorphism (SNP) near exon 1B called rs2004640, and a copy-number variant near exon 1A called rs77571059 (Figure 1). The rs77571059 polymorphism is an insertion/deletion (indel) of 5 bp, and is commonly referred to as a CGGGG indel. This study examines the promoters of IRF5, with information on how these two functional polymorphisms play a role in IRF5 expression. A general trend of these polymorphisms is to increase levels of IRF5.

The rs2004640 SNP is a G or T polymorphism near the 3′ end of exon 1B. The SNP is within the splice junction, such that when the G allele is present, the splice junction is not recognized and exon 1B cannot be spliced onto exon 2 (8). Unspliced transcripts are usually targeted by non-sense mediated decay (9). The risk T allele at this locus is associated with systemic lupus erythematosus (SLE) in multiple ethnic groups (8, 10–13) rheumatoid arthritis (14, 15), systemic sclerosis (16), multiple sclerosis (17), ulcerative colitis (18), and Sjögren’s syndrome (19). Autoimmune-risk haplotypes that include rs2004640 exhibit high IRF5 levels (6, 20), as well as high levels of IFNα and TNFα (21, 22).

The CGGGG indel (rs77571059) is found 64 bp upstream of the transcription start site for exon 1A. Each allele has either three (3×) or four copies (4×) of the CGGGG repeat sequence. The 4× copy-number variant allows binding of additional SP1 transcription factors (23). This 4× variant is associated with SLE (10), Sjögren’s syndrome (24), multiple sclerosis (17), Crohn’s disease and ulcerative colitis (18), and acute coronary syndrome (25). The CGGGG 4× variant is associated with increased expression of IRF5 itself (23), as well as TNFα, IL-12p40, IL-8, IL-1b, and IL-10 (22).

Interferon regulatory factor 5 exons 1A, 1B, 1C, and 1D each have a distinct transcriptional start site, and as is the case with every first exon, each exon 1 of IRF5 has its own promoter. IRF5’s four promoters have not been thoroughly characterized, although previous studies on the 1A and 1C exons’ promoters revealed that they are controlled in part by an IRF element (IRFE) and an interferon stimulatory response element (ISRE), respectively (26). Herein, we identify and characterize a putative promoter for exon 1B, and hypothesize that the 1B promoter would be active and regulated by stimuli that activate IRF5. We further hypothesize that the 1B promoter would be regulated by p53.

Autoimmune diseases are caused by environmental triggers in those with a genetic propensity. Increases in IRF5 expression due to these promoter polymorphisms could lead to an autoimmune-risk state. A hallmark of lupus and those at genetic risk for lupus is the presence of heightened levels of interferon and interferon-response genes; the interferon signature (27). IRF5 is a key gene in the interferon response to viral infection. IRF5 is a transcription factor whose activation leads to the interferon signature and the control of multiple genes involved in inflammation and immunity (28). It is primarily expressed in B cells, monocyte-derived cells, and plasmacytoid dendritic cells (pDCs) (2).

For SLE, an environmental trigger is likely to be Epstein–Barr virus (EBV) infection (29, 30). EBV infection affects IRF5 and IRF7 signaling, and has been associated with lupus through several different mechanisms (30–32). Interestingly, EBV infection alters IRF5 splicing to produce a dominant negative variant, suppressing the interferon response (33). For these studies, EBV-infected B cells are used, because cells with the appropriate genotypes can be immortalized and used in multiple experiments. As B cells, these cells are relevant to autoimmune disease and express IRF5. The incorporation of EBV into the model cells means that our results must be interpreted with caution, as it is possible that the major effects of these risk polymorphisms regulate or alter EBV infection, not IRF5 directly. These results must therefore be interpreted with caution. However, if it is found to be the case that these IRF5 polymorphisms affect EBV infection, that would likely provide even more exciting directions to pursue given the potential relationship between EBV infection and lupus.

## Results

### IRF5’s four promoters

**Figure 2:**
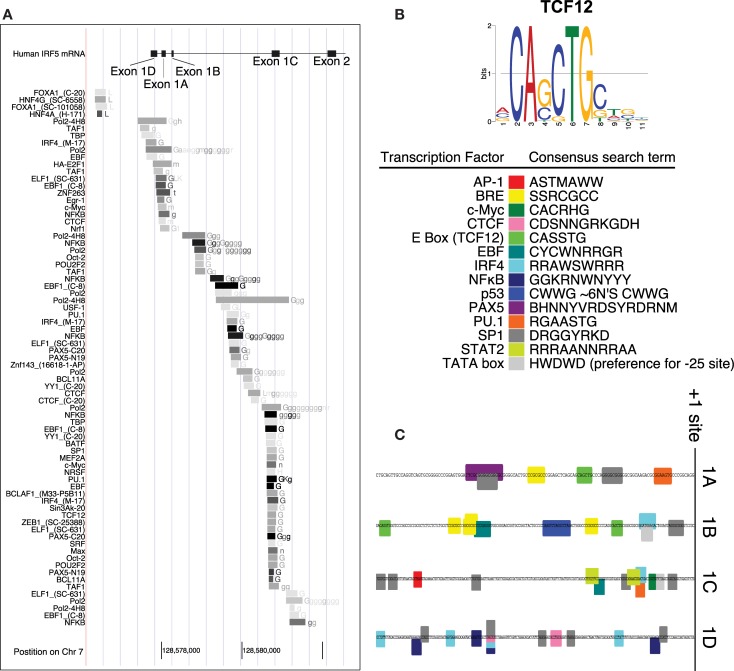
**Promoter analysis of each first exon of IRF5**. **(A)** ENCODE data shows results of ChIP-Seq analyses in the promoter region of IRF5. **(B)** The consensus search terms generated from FactorBook, with the TCF12 consensus binding site as an example (35). This data was used to manually define the nucleotide search terms shown. **(C)** The final analysis of potential binding sites is shown along the genomic DNA promoter sequences, with color-coded boxes representing the binding sites or transcription factors shown in the key. AP, activator protein; BRE, B-response element; CTCF, CCCTC binding factor; TCF, transcription factor; EBF, early B cell factor; IRF, interferon regulatory factor; NFκB, nuclear factor kappa light chain enhancer of activated B cells; PAX, paired box; PU, purine rich; SP, specificity protein; STAT, signal transducer and activator of proteins; TATA, thymidine adenine.

Interferon regulatory factor 5 uses one of four first exons for each molecule of mRNA – 1A, 1B, 1C, or 1D. Whether or not one of the four first exons of IRF5 would be actively transcribed depends on the cellular transcription factors that are able to bind it. A putative IRF5 exon 1B promoter sequence was identified by using the encyclopedia of DNA elements chromatin immunoprecipitation sequencing (ENCODE ChIP-Seq) data set (34). This analysis includes a list of transcription factors known to bind to the putative promoter sequence. An analysis of the promoters for each of the other three first exons of IRF5 was performed using the same database. This list represents results from many experiments which show transcription factors that bind to this genomic region of DNA (Figure 2A).

The transcription factors listed have also been associated with specific binding sites. WebLogos, which visualize consensus binding sites (36), were generated *de novo* for each ENCODE transcription factor tested, and compiled in the online database FactorBook (35). The consensus sites were converted manually into an ambiguous DNA code search term, where for example W (weak) represents an A or a T nucleotide (Figure 2B). The consensus search term was then used to search the proximal promoters (∼200 bp upstream from the +1 sites) to encounter a proposed binding site. Consensus search term screening was performed using MEGA (37).

Several transcription factors’ binding sites were found in the regions upstream of transcription start sites (Figure 1C). The start sites were taken from reference sequences for exons 1A, 1B, and 1C, and the sequence for variant 12 of IRF5 for exon 1D (no reference sequence exists at present for exon 1D). The source sequences are GenBank IDs NM_002200.3, NM_032643.3, NM_001098627.2, and EU258897.1 for exons 1A, 1B, 1C, and 1D, respectively. The workflow and results are shown in Figure 2, with transcription factor 12 (TCF12) as an example.

### Each IRF5 promoter exhibits transcriptional activity

The promoters for the four first exons of *IRF5* contain different potential transcription factor binding sites. The 1A promoter contains putative binding sites for paired box 5 (PAX5), PU.1, SP1, and TCF12 which binds to enhancer boxes (E boxes). An extra SP1 binding site appears in those with the CGGGG 4× indel. Exon 1B’s promoter was the only IRF5 promoter with a p53 binding site. This is discussed in more detail below. 1B also has SP1, TCF12, IRF4, and early B cell factor (EBF) sites. The 1C promoter was the only promoter with STAT2, activator protein 1 (AP1), and Myc binding sites; it also has SP1 and IRF4 sites. The 1D promoter evaluation showed potential binding sites for only four transcription factors: SP1, CCCTC binding factor (CTCF), IRF4, and NFκB.

To determine activity levels of each promoter, they were cloned using PCR and inserted into luciferase reporter plasmids. In addition to the 1B, 1C, and 1D promoters, there are two distinct versions of the 1A promoter, representing the two rs77571059 polymorphisms. One has the 4× variant of the CGGGG indel (1A_risk_), and the other has the 3× variant (1A_protective_). The 1B promoter was cloned using nested PCR to avoid an inverted repeat sequence located ∼2 kbp upstream. The inverted repeat is 1.8 kbp in length, and the two copies have 82.8% identity (34).

A luciferase assay was performed using the pGL4 plasmid. The promoters of IRF5 were inserted upstream of the luciferase gene and promoter activity was evaluated by measuring luminescence. The activity levels of the promoters were analyzed in several cell types since distinct transcription factors would be active in different cell types. Three types of immune cells were used: lymphoblastoid cell lines (LCLs), EBV-transformed human B cells that were generated from three healthy volunteers; U937 cells, a commercially available human monocyte cell line; and Jurkat cells, a commercially available human T cell line. Jurkat cells were used as the negative control, since T cells do not express high levels of *IRF5*. Cells were electroporated with each of the IRF5 promoter luciferase plasmids. A second plasmid, which expresses enhanced green fluorescent protein (eGFP), was cotransfected as a transfection control for each construct (38). Values for luciferase expression were then normalized to the fluorescence level to account for transfection efficiency.

**Figure 3:**
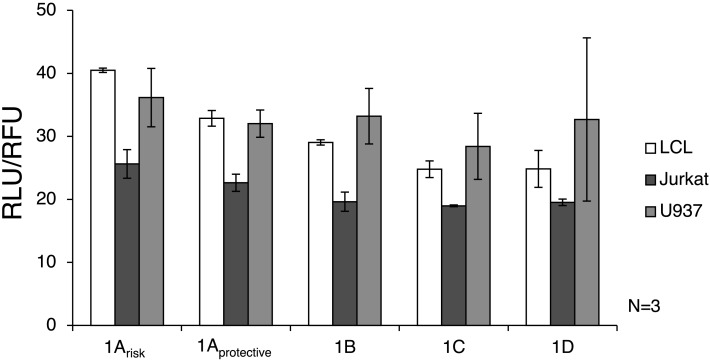
**Interferon regulatory factor 5 promoter activity in immune cells**. The luciferase plasmids were transfected by electroporation of three cell types: LCL, U937 cells, and Jurkat cells. A control GFP-encoding plasmid was also used in each sample to normalize transfection efficiency. ANOVA analysis revealed statistically significant variation between groups (*p* = 0.014); therefore *t*-tests were used to determine where the variation was found. The levels of transcription were significantly lower in Jurkat cells compared to LCL and U937 cells (*p* < 0.01). IRF5 is not highly expressed in T cells such as Jurkat cells, but is normally expressed in B cells and monocytes (39). The 1A promoters (1A_risk_ and 1A_protective_) displayed higher activity than the 1B, 1C, or 1D promoters in LCL and Jurkat cells (*p* = 0.0009 and *p* = 0.016, respectively). In LCLs the 1A_risk_ promoter activity was higher than 1A_protective_ promoter activity (*p* = 0.019). The putative 1B promoter acted like the 1A promoter in that expression was significantly higher in LCLs than in Jurkat cells (*p* = 0.027). LCL, lymphoblastoid cell line; RLU, relative luminescence units; RFU, relative fluorescence units. In some samples, the first exon was not detectable, which is why there is some variation in sample number.

Luciferase assay results showed that the 1A promoters (1A_risk_ and 1A_protective_) demonstrated significantly higher transcriptional activity than the other three promoters in LCL and Jurkat cells (*p* = 0.0009 and *p* = 0.016, respectively) (Figure 3). As expected, expression from all IRF5 promoters was significantly lower (*p* < 0.01) in Jurkat cells when compared to U937 or LCL cells. When comparing LCL to U937 cells, there was no significant difference in IRF5 promoter activity (*p* = 0.38).

The autoimmune-risk polymorphisms affected the activity of the promoters. In LCLs the 1A_risk_ promoter activity was significantly higher than 1A_protective_ promoter activity (*p* = 0.019). The 1B promoter, which is only relevant when the risk allele rs2004640 is present, showed activity in LCLs and U937 cells, indicating that it is an active promoter in the same cell types as the other promoters.

### The 1A and 1D promoters are affected by TLR7 ligation

The levels of IRF5 expression increase due to several signaling pathways, one of which is the Toll-like receptor 7 (TLR7) pathway. Endosomal TLRs such as TLR7 require the ligand to first be endocytosed into the cells, and then merged with the endosome that contains TLR7. Most endosomal TLRs bind to nucleic acids.

Toll-like receptor 7 ligation is an important method of activation for pDCs (40, 41). pDCs can produce large amounts of interferon alpha in response to immunostimulatory molecules such as nucleic acids. This is an important activation pathway in autoimmune disease (42). Single stranded RNA is the natural agonist for TLR7. TLR7 can also be activated by small synthetic compounds such as the imidazoquinolines, namely imiquimod and resiquimod. Imiquimod is a TLR7 ligand and resiquimod is a ligand for TLR7 and TLR8 (43). Imiquimod is used clinically as a topical cream as a form of treatment for genital warts and certain cancers. It activates the immune system, recruiting inflammatory mediators to kill the virus-infected or cancerous cells (44).

**Figure 4:**
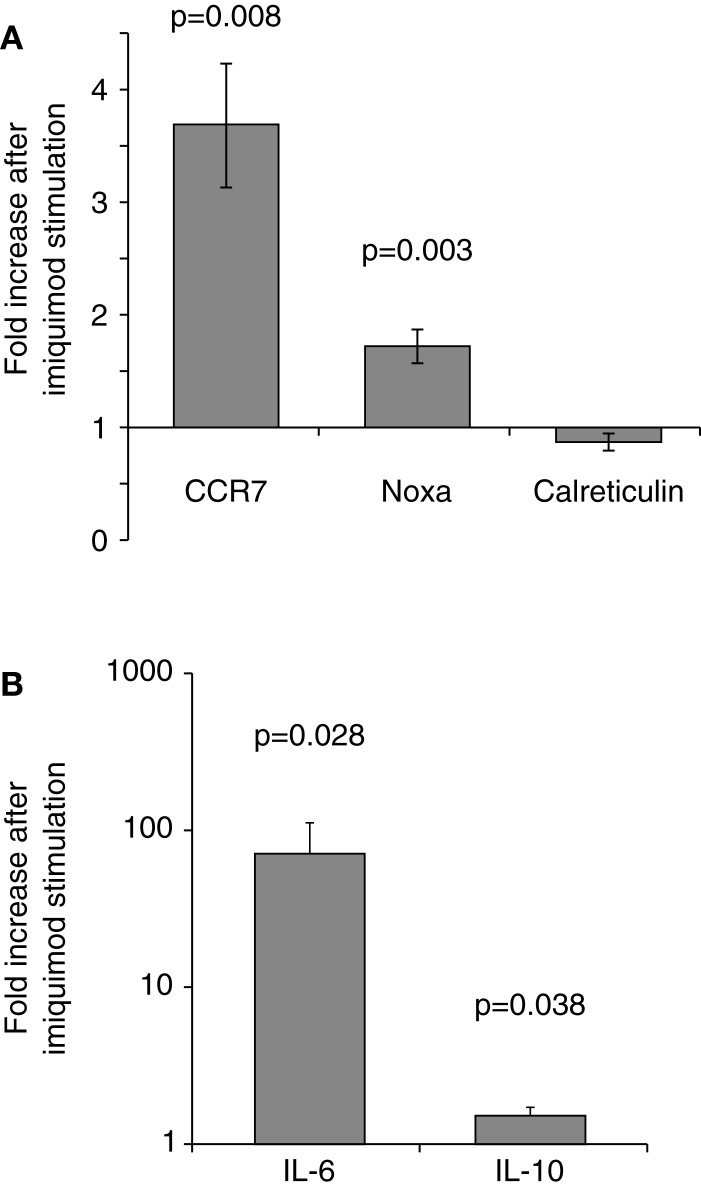
**Imiquimod stimulates LCLs to express cytokines and interferon-response genes**. LCLs were generated by EBV infection of cells from healthy volunteers. Cells were treated with imiquimod. **(A)** Expression of the interferon-response genes CCR7, NOXA, and Calreticulin were measured using SYBR Green real-time PCR. The figure shows the fold increase in gene expression after imiquimod stimulation for each gene. CCR7 and NOXA were significantly upregulated after imiquimod stimulation (*p* = 0.008 and *p* = 0.003, respectively). **(B)** Expression of cytokine RNA was measured using SYBR Green real-time PCR. IL-6 expression was upregulated by 71-fold after imiquimod stimulation (*p* = 0.028). IL-10 expression was also significantly upregulated (*p* = 0.038), although to a much lesser extent, at 1.5-fold. *N* = 12 for each experiment.

To verify that imiquimod treatment was stimulating the cells through TLR7, gene expression of interferon-response genes and cytokine gene expression were monitored using real-time PCR. Imiquimod stimulation led to significantly increased expression of the interferon-induced genes CCR7 and NOXA, while expression of the calreticulin was not significantly affected (Figure 4). Expression of the genes for the cytokine IL-6 was substantially upregulated (71-fold increase, *p* = 0.028). IL-6 expression is a common readout for stimulation through TLR7 (45, 46). Expression of the cytokine IL-10 is also significantly, though slightly, increased (1.5-fold, *p* = 0.038) after treatment with imiquimod (Figure 4). These results indicate that imiquimod treatment did in fact stimulate the cells.

**Figure 5:**
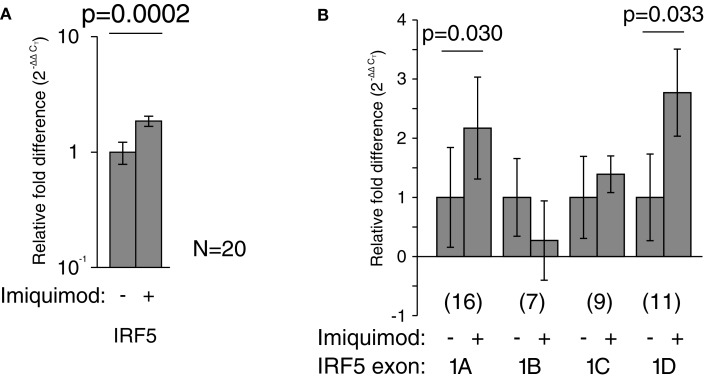
**Imiquimod caused increased IRF5 transcription through exons 1A and 1D**. All mRNA levels were measured in LCLs generated from healthy individuals. Levels were determined by TaqMan-based quantitative PCR using the 2^−ΔΔCT^ method. **(A)** The levels of IRF5 were 1.9-fold higher in treated cells (*p* = 0.0002). **(B)** The levels of exon 1A increased 2.2-fold (*p* = 0.030) and exon 1D increased by 2.8-fold (*p* = 0.033). All fold-increase values were normalized to the β-GUS housekeeping gene. The numbers in parentheses indicate the sample size. Analysis of variance was performed including each first exon and stimulation state as groups. This analysis revealed statistically significant variation (*p* < 0.0001). Statistical significance between individual groups was determined by paired *t*-test. IRF, interferon regulatory factor.

Imiquimod treatments were performed to determine the effects of stimulation on the activity of each IRF5 promoter. First exon-specific quantitative PCR was used to determine changes in the levels of each first exon after imiquimod stimulation. Cells were treated with imiquimod at 25 μg/ml for 24 h, and then cDNA was prepared from an RNA extract of treated cells. This was done for LCLs generated from 20 healthy individuals. As expected, IRF5 levels increased when cells were treated with imiquimod – a 1.9-fold increase when normalized to the housekeeping gene, β-glucuronidase (β-GUS) (Figure 5A).

The amounts of each first exon were also measured and compared to β-GUS by quantitative PCR. Several samples yielded undetectable levels of first exon transcripts, and were thus not included in the analysis, resulting in the variation in sample number noted in Figure 5. The levels of exons 1A and 1D increased by at least twofold after treatment with imiquimod when compared to β-GUS (Figure 5B).

**Figure 6:**
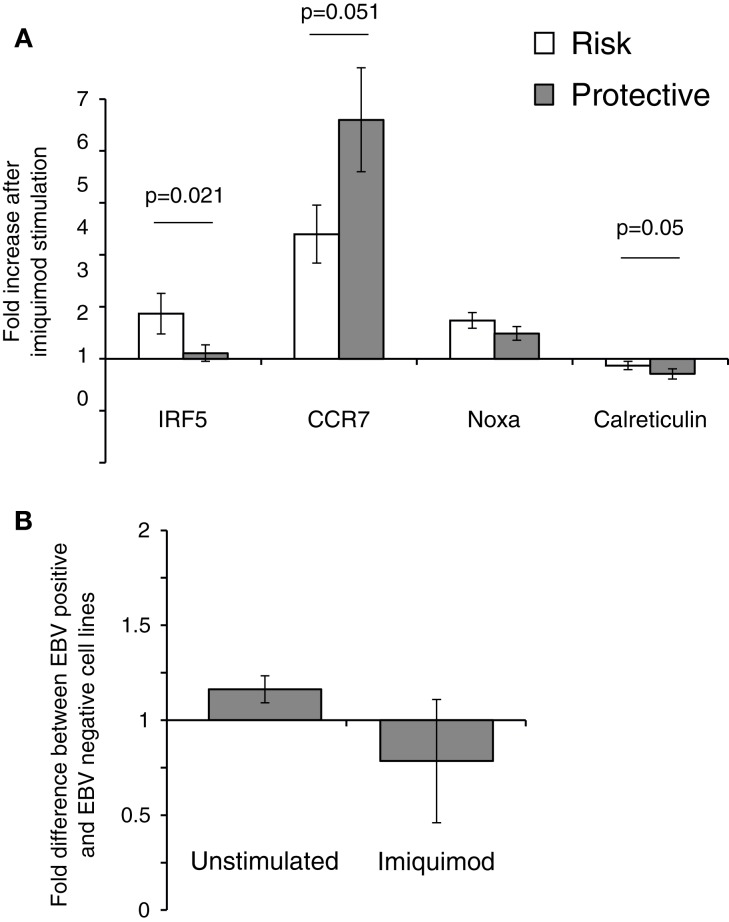
**The 2004640 risk allele affects responsiveness to TLR7 stimulation**. **(A)** LCLs with either risk or protective genotypes were treated with imiquimod to stimulate TLR7. Expression of interferon-response genes before and after stimulation was compared using SYBR Green real-time PCR. RNA input was normalized for each sample using the housekeeping gene GAPDH. Increase after imiquimod stimulation for risk and protective cells is shown. IRF5 expression is higher after imiquimod stimulation in the risk cells, by 1.67-fold (*p* = 0.021). Although Calreticulin expression decreases in both the risk and protective cells, it does so less in the risk cells (*p*-0.05). CCR7 increases less in the risk cells after imiquimod treatment (*p* = 0.05) *N* = 12 **(B)**. EBV status does not dramatically effect IRF5 levels. IRF5 expression was compared between LCLs and Ramos cells, a Burkitt’s lymphoma-derived B cell line that is EBV negative. IRF5 levels were not dramatically different between cell lines*N* = 2.

The effect of the rs2004640 polymorphism on imiquimod stimulation on was examined using real-time PCR. LCLs with risk or protective genotypes were stimulated with imiquimod, and the change in expression of interferon-stimulated genes was compared between risk and protective cells. IRF5 expression was higher in risk cells by nearly 1.7-fold (*p*-0.021). CCR7 did not increase as much after imiquimod stimulation in the risk cells compared to the protective (0.77-fold, *p* = 0.05). However, the risk cells demonstrated a small increase in NOXA expression after imiquimod stimulation, and the Calreticulin levels decreased less (by 1.35-fold, *p* = 0.05) in the risk cells than in the protective (Figure 6). These results show a small, but consistent increase in responsiveness to imiquimod stimulation in the cells with the risk allele.

The effect of EBV infection on IRF5 expression and imiquimod stimulation were analyzed. Ramos cells, a B cell line that is similar to LCL but is EBV negative, were stimulated with imiquimod and expression of IRF5 was compared to IRF5 expression in LCLs. After two experiments, there was a <1.2-fold difference in IRF5 expression between the EBV positive and EBV-negative cell lines. After imiquimod stimulation, there was similarly only a very small difference between the EBV positive LCL and EBV-negative Ramos cells (Figure 6B).

### The rs2004640 SNP’s role in p53 binding and activation

Mutagenesis of the 1B promoter p53 binding site did not alter transcriptional activity.

**Figure 7:**
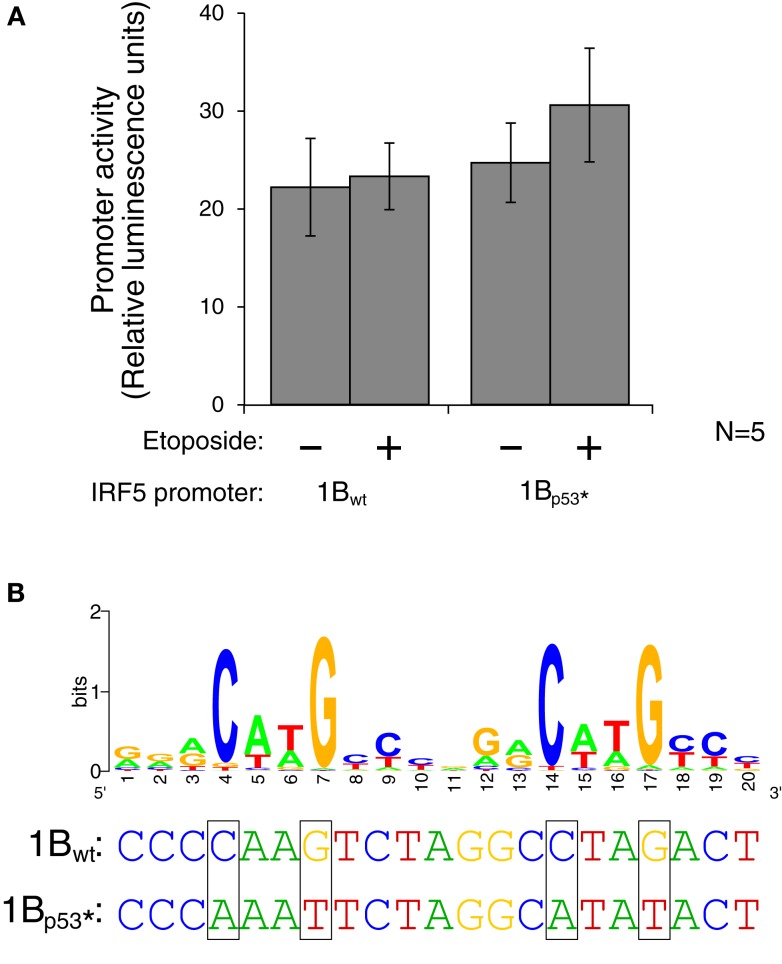
**Interferon regulatory factor 5 1B promoter activity is not regulated by direct p53 binding**. **(A)** Different LCLs generated from healthy volunteers were electroporated with the 1B_wt_ or the 1B_p53*_ promoter luciferase plasmid. Cells were treated with 0.5 mM etoposide or left untreated. Despite the presence of a putative p53 binding site, DNA damaging treatment did not affect promoter activity; neither did mutation of the p53 site. **(B)** The putative p53 binding site in IRF5’s exon 1B promoter, with a WebLogo of the p53 consensus binding site (29) to indicate important bases and matches. The height of the base represents the frequency of that nucleotide. Site-directed mutagenesis was performed to mutate the binding site at the critical C and G bases as shown. UV, ultraviolet; wt, wild type; *, mutant.

The promoter analysis described above revealed a potential p53 binding site. p53 binds as a tetramer to two copies of the sequence rrrCwwGyyy, with a spacer of 0–13 nt between the copies (189). A close match to this sequence was found in the 1B promoter (Figure 7B). This is suggestive because of the potential role IRF5 may play in apoptosis dysregulation in SLE. IRF5 is also proapoptotic in a p53-independent manner (47), and thus if p53 activates IRF5, apoptosis levels would be additively altered. Should p53 can control the 1B promoter, apoptosis would be altered in rs2004640 risk cells because the 1B promoter is only used in cells with the rs2004640 risk allele.

To test whether activated p53 protein can indeed bind to the p53 binding site, the plasmid which contains the 1B promoter was mutated using site-directed mutagenesis. Mutations were made to the wild-type sequence such that p53 should not be able to bind. The consensus binding site contains four conserved C or G bases which were mutated to A or T on the luciferase plasmid (Figure 7B). The wild type and p53-mutant luciferase plasmids were transfected by electroporation into three different LCLs generated from healthy volunteers. After 24 h to allow for plasmid expression, cells were either treated with etoposide or left untreated for 48 h. The levels of luciferase activity stayed fairly constant in the wild-type plasmid when treated with the etoposide. However, when the p53 binding site was mutated, thus inhibiting binding of p53, there is an slight, but non-significant increase in activity when treated with the etoposide versus being left untreated (Figure 7A). This finding suggests that if p53 does in fact bind to the IRF5 exon 1B promoter, it is likely inhibitory rather than stimulatory.

## Discussion

The CGGGG indel polymorphism within the 1A promoter has previously been shown to alter transcription factor binding. When cells have the 4× variant, an additional SP1 binding site is created. This has been shown to increase IRF5 in PBMCs (48), but decrease 1A-specific IRF5 transcripts in thymic cells (49). Both versions of the 1A promoter showed activity in HEK293T cells and Raji cells. As expected, SP1 sites were found in our analysis of the 1A promoter, including an extra SP1 binding site in those with the CGGGG 4× indel. SP1 is active during development, cell growth, apoptosis, differentiation, and immune and DNA damage responses (50).

The 1A promoter has a PAX5 binding site, a gene that activates B cells at early, but not late stages of development (51). There is an E box, and TCF12 is a member of the basic helix-loop-helix group of transcription factors which binds to E boxes (52). TCF12 was shown to bind somewhere in the promoter region of IRF5 in the ENCODE dataset (34), and the putative E box in 1A’s promoter is a likely site. TCF12 is known to be expressed in B cells and T cells (39). A PU.1 site is in the 1A promoter as well; PU.1 activates gene expression during B cell development and in myeloid cells (53).

The 1A promoter showed increased activity when cells were stimulated with the TLR7 agonist imiquimod. This may be through the PU.1 site through IRF7. IRF7 is known to be activated by TLR7 (54), and PU.1 binds to a similar GAAN_(N)_GAA motif to IRFs. Further work is necessary to determine in which cell types or with which stimuli the 1A promoter is most active, and in what instances the CGGGG 4× variant alters this activity.

A previous report by Mancl et al. evaluated the 1A and 1C promoters (26). The 1A promoter was activated by herpes simplex, Newcastle disease and vesicular stomatitis viruses in PBMCs, Daudi, and THP-1 cells; respectively; as evidenced by increased transcription of IRF5. A luciferase reporter gene assay also showed that IRF5’s 1A promoter is constitutively active and contains an IRFE consensus binding site. However, the promoter region used was a 596-bp region determined by a 5′ rapid amplification of cDNA ends (5′RACE) experiment and is 939 bp upstream of the GenBank reference sequences for exon 1A, and even extends past the 1D exon by 714 bp. The results of their luciferase assay cannot therefore be compared with the promoter analysis performed in this work. This work narrows the DNA regions studied and separates them into each of the four unique promoter elements, demonstrating that each are active promoters. This work also confirms that the 1A promoter is the strongest and is activated by imiquimod, and that the 1D promoter is also strongly activated by this stimulus.

The ability of a cell to use and splice the 1B exon is independent of its promoter usage. The 1B promoter is active in persons with both the risk and protective polymorphisms at rs2004640, yet the protective sequence would result in a non-sense transcript, as splicing would not be possible. The risk T allele allows for the exon 1B transcript to be spliced onto exon 2 and this is evidenced by the correlation between the risk T allele and increased levels of both IRF5 and exon 1B usage. The effects of the ability to use the 1B promoter can also be seen in the increased responsiveness of the cells containing the risk allele to imiquimod.

Interferon regulatory factor 5’s 1B promoter was predicted to contain a p53 binding site. The only promoter tested which increased in activity after inducing DNA damage was the 1B promoter. The others showed a reduction in luciferase activity (data not shown). The mutated version of the 1B promoter, which contained an altered p53 binding site, showed a slight increase in luciferase activity instead of a decrease, likely suggesting that any p53 binding to this promoter region is inhibitory. The 1B promoter contains SP1, IRF4, TCF12, and EBF binding sites. EBF is a B cell-specific transcription factor (55). Further work is necessary to reveal the stimuli or cell types that use the 1B promoter, as well as the combinations of transcription factors that drive transcription.

During cloning experiments dealing with exon 1B and its promoter, several sequencing reactions showed <100% sequence identity to the target. It was soon discovered that the primers were annealing to an upstream inverted repeat sequence. This repeat necessitated nested PCR for cloning the 1B promoter. The repeat length is 1.8 kbp, and the two copies have 82.8% identity (56). The function of this repeat is unknown, but repeated sequences can act as decoys for transcription factors, lowering transcription of the intended target (57).

Usage of exon 1C is lower in cells with the rs2004640 autoimmune-risk factor. The 1C promoter contains putative SP1, IRF4, and EBF sites. It was the only promoter with AP1, Myc, and STAT2 binding sites. AP1 is a heterodimer of Fos and Jun proteins, among others, which are common in immune signal transduction (58). Myc is a proto-oncogene, and is essential for B cell proliferation (59). STAT2, when complexed with STAT1 and IRF9, is known to be activated by type I interferon (60). The STAT2 binding sites agree with a previous report on the 1C promoter of IRF5 by Mancl et al. which said the promoter is interferon responsive (61). The current analysis identified the same STAT2 binding site in the 1C promoter. The difference in the two analyses is the assumed placement of the initiation site. The analysis by Mancl et al. uses 5′RACE to determine the initiation site and they calculate the STAT2 binding site is 96 bp downstream of that transcription initiation site. According to our initiation site – taken from the GenBank reference sequences which use exon 1C – the site was 47 bp upstream of the initiation site. Also of note, cells treated with imiquimod had lower 1C levels in proportion to the total IRF5.

Usage of exon 1D is lower in cells with the rs2004640 T allele and in cells with the CGGGG 4× allele. The 1D promoter evaluation showed only four transcription factors’ binding sites: CTCF, IRF4, NFκB, and SP1. NFκB is a target of TLR7 (62), and thus the promoter should be activated by imiquimod treatment. This was the case, and the 1D promoter nearly tripled in usage after imiquimod treatment. The IRF5 promoter analysis also showed a CTCF binding site. It is interesting that the 1D promoter is the furthest exon in the 5′ direction, and has putative CTCF sites, since CTCF is known to block the spread of CpG methylation by acting as an insulator (63). This may keep the other first exons – which are downstream and have high GC content – free from heterochromatin.

Interferon regulatory factor 5 is proapoptotic in a p53-independent manner (64, 65). This does not preclude modulation by p53, and a p53 enhancer site in exon 2 of IRF5 has been shown to activate IRF5 (66). p53 is a main regulator of apoptosis. Exon 1B’s promoter was the only one with a putative p53 binding site, and cells with the rs2004640 risk T allele are the only cells that can use exon 1B. Also, p53 can act as both a repressor and activator of transcription depending on local factors (67). However, in our assay, p53 did not significantly regulate the 1B promoter.

Epstein–Barr virus infection is a necessary complicating factor when using LCLs as B cell lines. This is especially important since EBV has been shown to affect IRF5 function (33, 68). The effect of EBV in this study was limited by using EBV-infected LCLs as both our risk and protective cell lines. Since cell lines of both genotypes are transformed with EBV, the differences observed should be comparable and the effect of EBV excluded. However, given the importance of EBV infection in IRF5 activity and the development of lupus, viral effects cannot be simply discounted. There is a chance that differential effects of the risk polymorphisms on EBV infection processes are affecting IRF5 activity. These effects would be difficult to differentiate from direct effects on IRF5 activity. Either way, however, the results would be interesting and merit further investigation.

Autoimmune diseases are complex, multifactorial disorders with both genetic and environmental influences. The promoter variations examined in these experiments are strongly linked to risk for autoimmune diseases, including lupus, multiple sclerosis, and rheumatoid arthritis. Despite much effort, there has not been a dramatic effect associated with these polymorphisms, or really, most of the polymorphisms associated with autoimmune disease. Rather than diminishing their importance, however, the somewhat small effects observed here speak to the fine balance of the immune system. It is likely that even relatively small changes in gene regulation can lead to an imbalance in tolerance or activation of immune cells. Also, these genes are intertwined with other pathways and systems to provide a complex fabric controlling the level of immune responsiveness.

## Materials and Methods

### Plasmid construction and luciferase assay

All vectors were sequenced to confirm the proper sequence. The plasmid pMax-GFP (Clontech) expresses the eGFP fluorescent protein, and it was used to measure transfection efficiency. Electroporations were performed using a Nucleofector device (Lonza). The electroporation buffer was 5 mM KCl, 15 mM MgCl_2_, 15 mM HEPES, 140 mM Na_2_HPO_4_, pH 7.2. Transfected cells were lysed and assayed for fluorescence levels before assaying luciferase activity using the Luciferase Assay System (Promega) on a Fusion αHT plate reader (Packard). Luciferase activity was evaluated in proportion to the transfection efficiency.

### Cell lines

Peripheral blood samples were obtained from healthy volunteers after informed consent following a protocol approved by the IRB at Brigham Young University. Peripheral blood mononuclear cells were isolated using lymphocyte separation medium (Mediatech). These cells were induced to form LCLs by incubation with EBV (B95-8 strain) and 2 ng/ml cyclosporin A (Tocris Biosciences). U937 and Jurkat cells were a kind gift from Dr. Kim O’Neill. Cell lines were maintained in RPMI (Sigma) with 10% fetal bovine serum (PerBio) with penicillin/streptomycin/amphotericin (Calbiochem) at 5% CO_2_ and passaged at least weekly.

### Genotyping of volunteers and formation of paired samples

Genomic DNA was extracted (Qiagen) from peripheral blood mononuclear cells and genotyped using TaqMan reagents Applied Biosystems (ABI) on a StepOnePlus real-time PCR machine (ABI) at the rs2004640 SNP (ABI SNP Assay C9491614). Homozygous risk or protective individuals were matched by gender and ethnicity. Heterozygotes were not included in the study. The primers and PCR conditions are in Table A1 in Appendix.

### Cell treatments

The TLR7 ligand imiquimod (R-837) was used to stimulate cells for some experiments. Cells were treated for 24 h with 25 μg/ml imiquimod (InvivoGen). cDNA preparation, quantitative PCR, primers, probes, and conditions are described elsewhere in the Section “Materials and Methods.” Etoposide was used at 0.1 and 1 mM concentrations and applied for 48 h. 5FU was used at 1.5 mg/ml, and the activating antibodies to TRAIL and Fas were used at 1 and 5 μg/ml, respectively. All treatments used 10^6^ cells per milliliter.

### cDNA libraries and PCR

About 8 × 10^6^ cells were used for each condition in each experiment. cDNA preparations were made by extracting RNA using the RNaqueous system (Ambion), followed by DNase treatment (Promega). One thousand nanograms RNA was per condition was then reverse transcribed using SuperScript III reverse transcriptase (Invitrogen Life Technologies). One hundred nanograms cDNA preparations were used as template for quantitative PCR using TaqMan reagents (ABI), or SYBR green reagents For gene expression studies, input RNA levels were normalized using primers to the housekeeping gene GAPDH for SYBR green experiments and β-GUS for TaqMan experiments. For cloning of 5′UTRs and promoters the template genomic DNA from Section “Genotyping of Volunteers and Formation of Paired Samples” was used, with the NEB High GC PCR kit. Primers were purchased from Integrated DNA Technologies. Sequences and PCR conditions are available in Table A1 in Appendix.

### Sequencing

Plasmid sequencing used purified plasmid DNA and a primer upstream of the insertion site. Sequencing reactions used Big Dye terminator reagents and the 3730xl DNA analyzer (ABI). See Table A1 in Appendix for primers.

### Statistical analysis

A paired *t*-test was used to compare means for mRNA expression. Paired *t*-test was used for luciferase levels. An alpha value of 0.05 and two-tailed *p* values were used in all cases. For experiments using more than two comparisons, ANOVA was used to determine if statistically significant differences were present. Statistical analysis was performed using Data Analysis Plus software (Keller Statistics). ANOVA was performed using the CSBJU online calculator (http://www.physics.csbsju.edu/stats/).

### Promoter analysis

An analysis of the promoters for each of the four first exons of IRF5 was performed using the ENCODE ChIP-Seq data set (34) for determining actual binding factors on the genomic region, followed by determining a consensus site using the WebLogo data in FactorBook (35). The consensus site was then used to search the proximal promoters (∼200 bp upstream from the +1 sites) to encounter a proposed binding site. Consensus site screening was performed using a custom searches of ambiguous nucleotides with MEGA (37). This involved searching using the find function, which allows for searching using the ambiguous nucleotide code. For example, a search for GAW would highlight both GAA and GAT.

## Conflict of Interest Statement

The authors declare that the research was conducted in the absence of any commercial or financial relationships that could be construed as a potential conflict of interest.

## Appendix

**Table A1:** **List of primers and PCR conditions**.

**TAQMAN-BASED QUANTITATIVE PCR PRIMERS**
IRF5 exon 2 RT fwd: CCACCTCAGCCCTACAAGAT
IRF5 probe: FAM-TCCAATGGCCCTGCTCCCAC-TAMRA
IRF5 exon 3 RT rev: CTCCTCTCCTGCACCAAAAG
IRF5 1A TaqMan RT fwd: ACGCAGGCGCACCGCAGACA
IRF5 1B RT fwd: AGCTGCGCCTGGAAAGCGAGC
IRF5 1C TaqMan RT fwd: AGGCGGCACTAGGCAGGTGCAAC
IRF5 1D RT fwd: GAGGCTCAGCCCGGATCTGC
IRF5 exon 1 probe: FAM-CCATGAACCAGTCCATCCCAGTGGCTCCCACC- TAMRA
IRF5 exon 2 common RT rev: TCGTAGATCTTGTAGGGCTGAGGTGGCA
β-Glucuronidase fwd: CTCATTTGGAATTTTGCCGATT
β-Glucuronidase probe: FAM-TGAACAGTCACCGACGAGAG-TAMRA
β-Clucuronidase rev: CCGAGTGAAGATCCCCTTTTTA
Conditions: 52°C, 95°C for 10 min, 52 cycles of (95°C for 15 s, 65°C* for 1 min) with 500 nM primers, 250 nM probe
**SYBR green quantitative PCR primers and conditions**
GAPDH fwd: TGCACCACCAACTGCTTAGC
GAPDH rev: GGCATGGACTGTGGTCATGAG
CCR7 fwd: GCTCCAGGCACGCAACTT
CCR7 rev: GACCACAGCGATGATCACCTT
Calreticulin fwd: GCAGCAGAAGGGGGTGGTGT
Calreticulin rev: GTCCTGGGGGCAGGGGAGAA
NOXA fwd: GCTGTCCGAGGTGCTCCAGTT
NOXA rev: AGCGTTCTTGCGCGCCTTCT
IRF5 fwd: CCACCTCAGCCCTACAAGAT
IRF5 rev: CTCCTCTCCTGCACCAAAAG
Conditions: 95°C for 10 min, 40 cycles of (95°C for 15 s and 60°C for 1 min)
**PROMOTER CLONING PRIMERS^†^**
IRF5 1A prom fwd: CTGCgctagcCAGGTCAGTGCGGGGC
IRF5 1A prom rev: CCTGagatctACTTCCGCGTCTTGCCGC
Conditions: 94°C for 30 s, 40 cycles of (94°C for 15 s, 62.0°C for 1 min, 68°C for 30 s), 68°C for 5 min
IRF5 1B prom fwd: GCGCgctagcGACAGGTGGGTCCCGGCCGC
IRF5 1B prom rev: GCAGagatctGCGGACCCCGCCCTACTCCA
Nested PCR first round: IRF5 1A prom fwd + IRF5 1B prom rev
Conditions: 94°C for 30 s, 40 cycles of (94°C for 15 s, 59.3°C for 1 min, 68°C for 30 s), 68°C for 5 min
Nested PCR second round: IRF5 1B prom fwd + IRF5 1B prom rev
Conditions: 94°C for 30 s, 40 cycles of (94°C for 15 s, 66.0°C for 1 min, 68°C for 30 s), 68°C for 5 min
IRF5 1C prom fwd: TAGTgctagcGCTGGTTTCCTCAGGTCCT
IRF5 1C prom rev: CAGAagatctCAGCCCTGCCCTGGCCT
Conditions: 94°C for 30 s, 40 cycles of (94°C for 15 s, 60.8°C for 1 min, 68°C for 2 min), 68°C for 5 min
IRF5 1D prom fwd: ACATgctagCACCTGCTGCCTGTTGACC
IRF5 1D prom rev: TGGCagatctGTCATTTGACAACCCC
Conditions: 94°C for 30 s, 40 cycles of (94°C for 15 s, 59.4°C for 1 min, 68°C for 1 min), 68°C for 5 min
pGL4 sequencing fwd: CTAGCAAAATAGGCTGTCCC

**Primer annealing temperatures were 60°C for β-glucuronidase; 65°C for IRF5, 1A, and 1B; 66°C for 1C and 69°C for 1D*.

*^†^PCR for these GC-rich promoters was performed using a high-GC kit (NEB) according to package instructions, with 10% enhancer solution included for all reactions except exon 1D*.

*All are listed in 5′ to 3′ orientation. Restriction enzyme cut sites or overhangs are indicated in lowercase. FAM, fluorescein amidite; IRF5, interferon regulatory factor 5; RT, real time; TAMRA, carboxytetramethylrhodamine*.
